# Self-regeneration of supported transition metals by a high entropy-driven principle

**DOI:** 10.1038/s41467-021-26160-8

**Published:** 2021-10-11

**Authors:** Shengtai Hou, Xuefeng Ma, Yuan Shu, Jiafeng Bao, Qiuyue Zhang, Mingshu Chen, Pengfei Zhang, Sheng Dai

**Affiliations:** 1 School of Chemistry and Chemical Engineering, Shanghai Jiao Tong University, Shanghai, 200240 China; 2 State Key Laboratory of Physical Chemistry of Solid Surfaces, National Engineering Laboratory for Green Chemical Productions of Alcohols-Ethers-Esters, Department of Chemistry, College of Chemistry and Chemical Engineering, Xiamen University, Xiamen, 361005 China; 3 Chemical Sciences Division, Oak Ridge National Laboratory, Oak Ridge, TN 37831 USA

**Keywords:** Solid-phase synthesis, Materials chemistry, Porous materials

## Abstract

The sintering of Supported Transition Metal Catalysts (STMCs) is a core issue during high temperature catalysis. Perovskite oxides as host matrix for STMCs are proven to be sintering-resistance, leading to a family of self-regenerative materials. However, none other design principles for self-regenerative catalysts were put forward since 2002, which cannot satisfy diverse catalytic processes. Herein, inspired by the principle of high entropy-stabilized structure, a concept whether entropy driving force could promote the self-regeneration process is proposed. To verify it, a high entropy cubic Zr_0.5_(NiFeCuMnCo)_0.5_O_x_ is constructed as a host model, and interestingly in situ reversible exsolution-dissolution of supported metallic species are observed in multi redox cycles. Notably, in situ exsolved transition metals from high entropy Zr_0.5_(NiFeCuMnCo)_0.5_O_x_ support, whose entropic contribution (TΔS_*config*_ = T⋆12.7 J mol^−1^ K^−1^) is predominant in ∆G, affording ultrahigh thermal stability in long-term CO_2_ hydrogenation (400 °C, >500 h). Current theory may inspire more STWCs with excellent sintering-resistance performance.

## Abstract (web summary)

Sintering of supported transition metal catalysts is problematic for high-temperature catalysis. Here the authors report entropy-driven, self-regeneration of high entropy oxide nanoparticles, promoting better stability and in situ reversible exsolution-dissolution process in multi-redox cycles.

## Introduction

Supported metal catalysts are of great importance in both industrial and fundamental catalysis^1^. The methods for generating active sites on metal oxide carriers include chemical or physical deposition techniques (e.g., impregnation method^2^, deposition-precipitation method^3^, co-precipitation process^4^, atomic layer deposition^5^, spraying^6^ and so on^7^. Needless to say, those methods have significantly promoted the synthetic chemistry of supported metal catalysts. For example, the control of metal nanocrystal size by modified deposition method reveals the attractive role of corner atoms, which offers a theoretical guidance for catalyst design^8^. Nevertheless, the deactivation of supported metal catalysts, especially those metal nanoparticles (NPs) with low tamman temperatures (e.g., Cu^9^, Ag^10^, Au^11^) and high surface energies^12^, is often inevitable by sintering^13^ under long-term exposure to the high temperature or redox environments. For example, Au/TiO_2_ catalysts afforded an ultralow temperature for CO oxidation^14^, while particle growth leading to the activity loss often occurred during high temperature operation^15^.

**Fig. 1:**
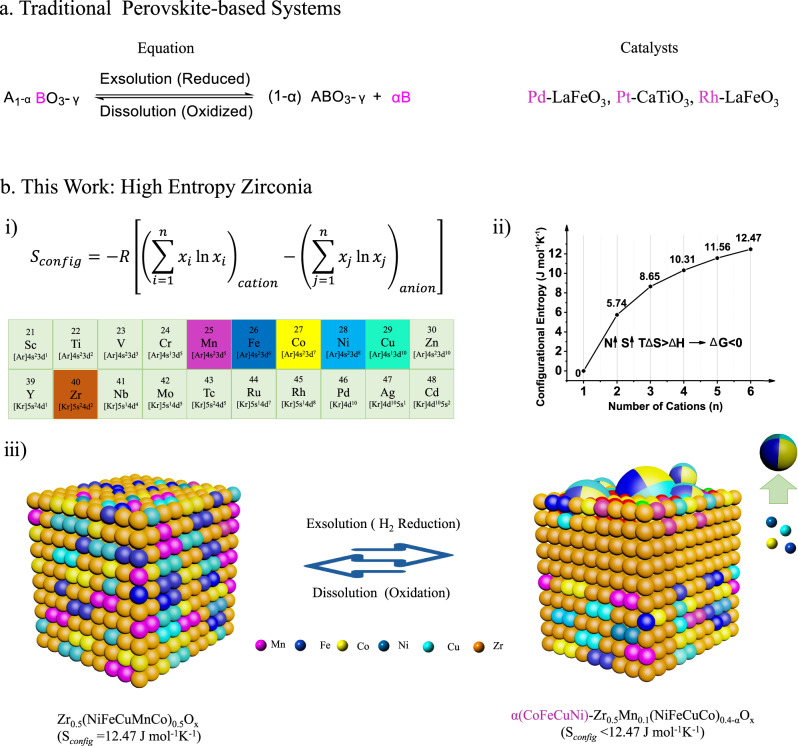
Comparison of self-regenerative materials. **a** Representative perovskite-based self-regenerative materials. **b** i. The configurational entropy formula, and the selected metals for high entropy ZrO_2_. ii. Calculated values of configurational entropy for Zr_0.5_(M_1_-M_n_)_0.5_O_x_, *n* = 1–5 (for example, n = 2, Zr_0.5_(M_1_M_2_)_0.5_O_x_). iii. Schematic representation of in situ exsolution and dissolution of CoFeCuNi alloys in the Zr_0.5_(NiFeCuMnCo)_0.5_O_x_ during redox processes. For simple, oxygen ions were omitted.

Early in this century, a self-regenerative strategy by in situ exsolution of active species from host matrix was presented by Nishihatas’ group^16^, which can greatly prolong the lifetime of three-way catalysts. The emergence of perovskite-based (ABO_3_) self-regenerative catalysts exploits a useful approach to optimize catalyst activity and stability^16–21^. To our knowledge, the control of nonstoichiometry^18^ (A-site defect interactions), judicious choice of B-site composition (e.g., Pd^17^, Pt^22^, Ni^23^, CoFe alloy^24^) and the surface modification (polished or pre-treated perovskite) are frequently used to tailor B-site metals exsolution. This type of catalytic materials can not only in situ generate active sites, but also accomplish reintegration of those species back to parent perovskites during oxidation-reduction cycles^16–21^. Meanwhile, introducing high-valence cations (Nb^25^, Ti^26^, Ga^27^, etc.) into ABO_3_ structure can improve the structural stability of the parent perovskites, which contributes to the reintegration process during catalysis^19^. This process effectively suppresses the sintering or agglomeration of the supported metals^16^. However, these types of host matrix are limited to perovskite crystal structures and the state-of-art self-generation systems are perovskite-based catalysts^16–19^ (Fig. 1a), which cannot meet the broad requirements by different catalytic processes. To date, the proposal of a self-regenerative principle for the design of sintering-resistance catalysts is still desired.

Since the discovery of high entropy alloys in 2004^28^ and the pioneering introduction of high-entropy oxides (HEOs: oxide solid solutions contain five or more distinct metal cations) in 2015^29^, entropy-stabilized materials such as oxides^30^, sulfides^31^, phosphide^32^, nitrides^33^, carbides^34^, and borides^35^, are flourishing in the library of advanced materials during past five years. Inspired by the principle of thermodynamics^29,30^ (Entropic contribution could predominate the thermodynamic landscape), applying the theory of high entropy materials to trigger the self-regeneration during catalytic redox processes may be a choice. First, based on the configurational entropy^34^ (it refers to the number of conformations of a molecule and the number of ways that atoms or molecules pack together in the oxide) formula (Fig. 1.bi), more disorder of system and higher randomness of structure with a lower Gibbs free energy can contribute to the stability of host structure, especially during high temperature catalysis (ΔG = H-TΔS)^31,36^ (Fig. 1bii). From another perspective, the reintegration process can be promoted due to an entropy-added motivation by the oxidative transformation of isolated metals back into parent metal oxides. The theoretical conjecture stimulates us to carry out a number of representative experiments.

In this work, we verify the entropy-driven principle for self-regenerative catalyst by high entropy ZrO_2_. A single-phase cubic Zr_0.5_(NiFeCuMnCo)_0.5_O_x_, whose entropic contribution (TΔS_*config*_ = T*12.7 J mol^−1^ K^−1^) is predominant in ∆G^28,29,31^, was prepared and rationally selected as a model catalyst. Interestingly, reductive H_2_ treatment could induce the exsolution of CoFeCuNi alloy NPs on the surface of parent high entropy ZrO_2_ NPs. With high entropy Zr_0.5_(NiFeCuMnCo)_0.5_O_x_ as the host matrix, the exsolution and reintegration of CoFeCuNi alloy were observed during three redox cycles (H_2_ 600 °C + Air 550 °C), an interesting feature that may confine metal species under dynamics catalysis (Fig. 1biii). As a proof of principle, the entropy-driven self-regenerative property endows Zr_0.5_(NiFeCuMnCo)_0.5_O_x_ catalyst good stability during CO_2_ hydrogenation reaction. The Zr_0.5_(NiFeCuMnCo)_0.5_O_x_ catalyst functioned well at 400 °C during 500 h continuous hydrogenation, and at the same time no phase separation or sintering were found on the reused catalyst. The self-regenerative talent of high entropy material may open an alternative for the design of sintering-resistance catalysts.

## Results

### The formation of high entropy cubic Zr_0.5_(NiFeCuMnCo)_0.5_O_x_

**Fig. 2:**
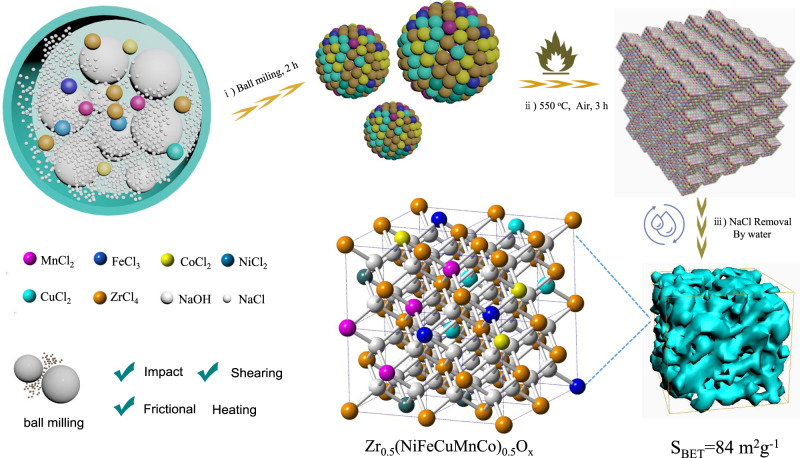
The synthetic route of entropy-stabilized Zr_0.5_(NiFeCuMnCo)_0.5_O_x._ i Mixing metal chlorates ZrCl_4_, MnCl_2,_ NiCl_2_, FeCl_3_, CoCl_2_, CuCl_2_, NaCl and NaOH by ball milling. ii Calcination of the intermediate in air. iii Sodium salt removal from the composite by washing.

Inspired by the principle of the high entropy-stabilized structure, the synthesis of high entropy ZrO_2_ catalyst was carefully investigated by a mechanochemical process (Fig. 2). Five common transition metal salts (Ni, Fe, Cu, Mn, Co) were mixed with ZrCl_4_ and NaOH, followed by calcination in air and sodium salt removal. Definitely, for transition metal dopants, the matching radius (the rationally selected five metals ${{\mbox{|}}}\frac{{{{\mbox{r}}}}_{{{\mbox{Zr}}}}{{\mbox{-}}}{{{\mbox{r}}}}_{{{\mbox{x}}}}}{{{{\mbox{r}}}}_{{{\mbox{Zr}}}}}{{\mbox{|}}}$ < 20%^37^: Ni, Fe, Cu, Mn, Co) was a crucial factor for achieving the highly doped ZrO_2_ solid solution. Binary doped ZrO_2_ samples were initially prepared. Impure phases were found (Supplementary Fig. 1) in Zr_0.5_Cu_0.5_O_x_, Zr_0.5_Mn_0.5_O_x_, Zr_0.5_Co_0.5_O_x_, and Zr_0.5_Ni_0.5_O_x_, presumably due to the limited solubility of the dopants in ZrO_2_^38^. At current stage, it is still difficult to incorporate 50 mol% dopants into monocrystalline ZrO_2_^39^. In addition, a series of Zr_0.9_M_0.1_O_x_ (M: Co, Ni, Mn, Cu, Fe) materials were constructed by the same synthesis conditions. Meanwhile, the doped metal content is the same as Zr_0.5_Ni_0.1_Fe_0.1_Cu_0.1_Mn_0.1_Co_0.1_O_x_ sample. Deserved to be mentioned, the Zr_0.9_Cu_0.1_O_x_, Zr_0.9_Ni_0.1_O_x_ exhibited obvious impurity peaks, which could be ascribed to the CuO, NiO, respectively (Supplementary Fig. 2a, b). It seems reasonable that 10% Cu-doped and 10% Ni-doped ZrO_x_ materials would present obvious diffraction peaks of NiO and CuO. The enhancement of entropic contribution may compensate the formation enthalpy^40^, and then a detailed study for constructing high entropy Zr_0.5_(NiFeCuMnCo)_0.5_O_x_ was carried out.

**Fig. 3:**
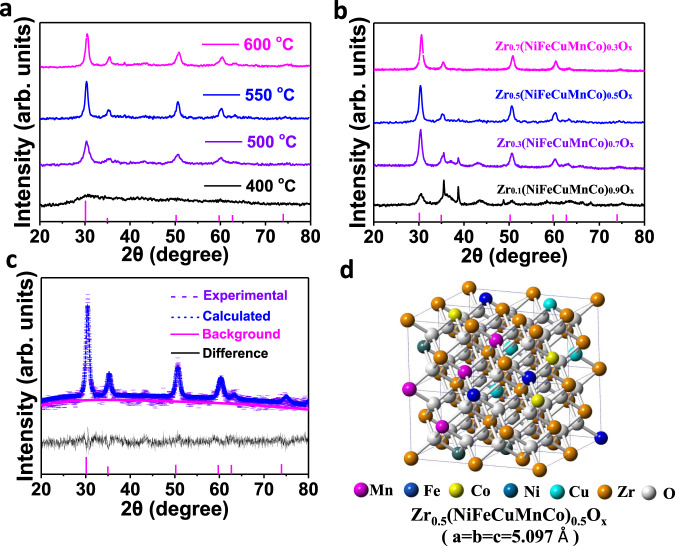
The synthesis of a cubic phase Zr_0.5_(NiFeCuMnCo)_0.5_O_x_. **a** The phase evolution of Zr_0.5_(NiFeCuMnCo)_0.5_O_x_ precursors under different calcination temperatures. **b** The P-XRD patterns of ZrO_2_ doped with different total ratio (0.3–0.9) of five metals. **c** P-XRD patterns together with Rietveld fits of the Zr_0.5_(NiFeCuMnCo)_0.5_O_x_ sample prepared at 550 °C. **d** Structural view of cubic Zr_0.5_(NiFeCuMnCo)_0.5_O_x_. arb. units: arbitrary unit.

Firstly, the calcination temperature was adjusted to explore the phase evolution of Zr_0.5_(NiFeCuMnCo)_0.5_O_x_ precursors. As shown in the Fig. 3a, obvious diffraction peaks for the typical cubic ZrO_2_ phase started to form at 550 °C. Notably, no other impurity peaks were observed at an elevated temperature (e.g., 600 °C). This phenomenon indicated the possible formation of a high entropy cubic Zr_0.5_(NiFeCuMnCo)_0.5_O_x_. Then, the solubility of five metals (Ni, Fe, Cu, Mn, Co) within ZrO_2_ solid solution was carefully investigated by adjusting their total ratio (0.3–0.9) (Fig. 3b). The single cubic structure of ZrO_2_ was well maintained in Zr_0.7_(NiFeCuMnCo)_0.3_O_x_ and Zr_0.5_(NiFeCuMnCo)_0.5_O_x_, while impure reflections of other crystal structures were detected with a higher ratio (0.7–0.9) of doped metals (Fig. 3b). The molar ratio (Zr:Ni:Fe:Cu:Mn:Co= 5:1:1:1:1:1) was then fixed, and a relative high configurational entropy (S_*config*_ = 12.47 J mol^−1^ K^−1^) could be expected.

Rietveld refinement was then performed to determine the structural parameters of Zr_0.5_(NiFeCuMnCo)_0.5_O_x_. The initial structural model was built based on the cubic ZrO_2_ (*a* = *b* = *c* = 5.100 Å). The calculated lattice constant of Zr_0.5_(NiFeCuMnCo)_0.5_O_x_ was *a* = *b* = *c* = 5.097 Å (Fig. 3c, d). The difference in atomic sizes between Zr and transition metals would cause the shrinkage of the lattice of ZrO_2_ matrix^41^. However, it should be noted that the abundance of oxygen vacancies in doped ZrO_2_ may enlarge the ZrO_2_ lattice^42^. Meanwhile, the R-factor (Rp (%)=4.6), weighted profiles R-factor (Rwp (%)=4.7) and chi square (*χ*^2^ = 1.330) of the HE ZrO_2_ were quite good (Fig. 3c). The simulated PXRD pattern of the high entropy ZrO_2_ was in good agreement with the experimental one. Those XRD results suggest the formation of cubic Zr_0.5_(NiFeCuMnCo)_0.5_O_x_.

With the option temperature (550 °C) and composition in hand, a high specific surface area (84 m^2^ g^−1^) was obtained by optimizing the dosage of NaCl additive (Supplementary Table 1. Supplementary Fig. 3). To the best of our knowledge, the high temperature for phase transformation (e.g., 900–1300 °C) in entropy-driving force (TΔS) would cause the collapse of pores, leading to HEOs with low surface areas, such as, NiMgCuZnCoOx by solid-state combustion (2 m^2^ g^−1^)^43^, NiMgCuZnCoO_x_ by citric acid-based sol–gel method (28 m^2^ g^−1^)^43^, (MgTiZnCuFe)_3_O_4_ by solid-state combustion (12 m^2^ g^−1^)^44^, CoNiCuMgZnO_x_ with graphene oxide as a sacrificial template (42 m^2^ g^−1^)^45^. The current porosity (84 m^2^ g^−1^) may be attributed to the particle breakage by mechanochemistry^46^, and meanwhile a mild crystallization temperature (550 °C) could effectively avoid the excessive growth of fine NPs and interstitial porosity was retained^47^.

**Fig. 4:**
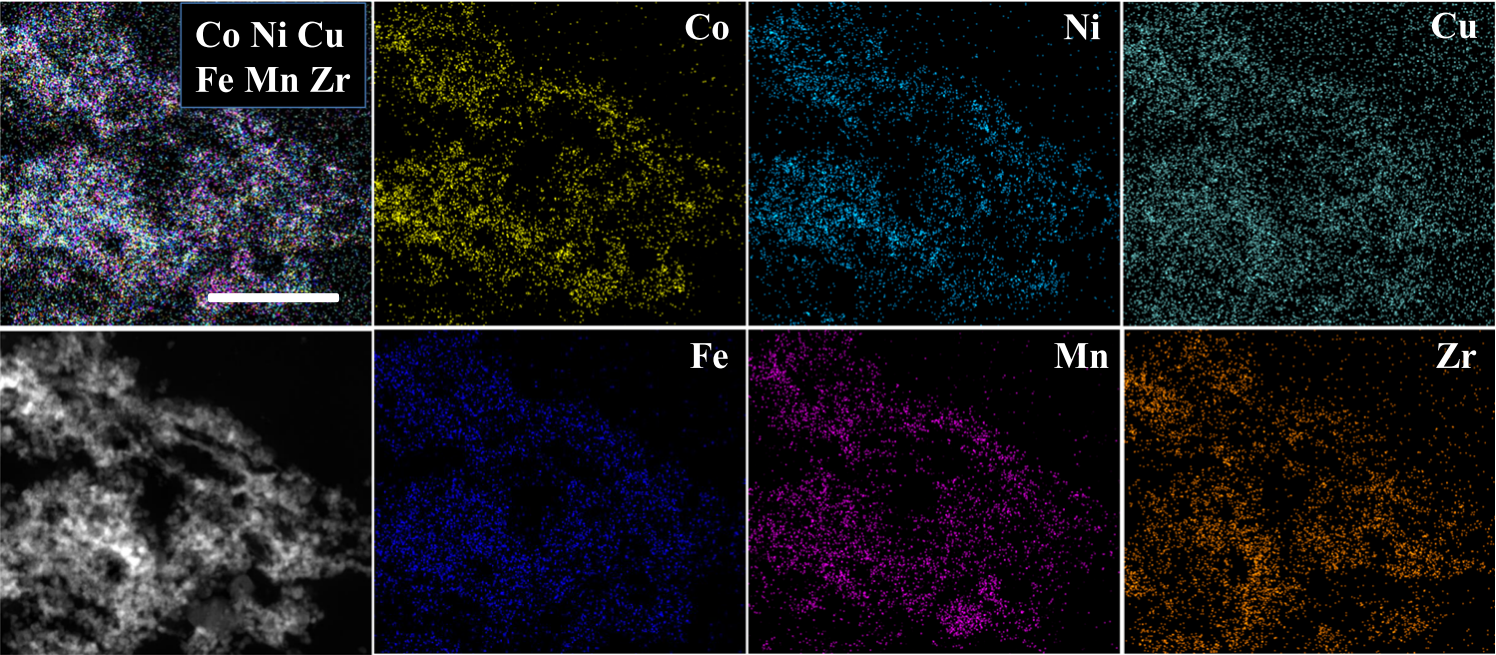
The characterization of freshly made Zr_0.5_(NiFeCuMnCo)_0.5_O_x_. STEM**-**HAADF image and EDS elemental maps of Zr_0.5_(NiFeCuMnCo)_0.5_O_x_. Scale bar, 200 nm.

In order to observe the morphology of the Zr_0.5_(NiFeCuMnCo)_0.5_O_x_ sample, the high-angle annular dark-field signal (HAADF) and energy dispersive spectrometer (EDS) elemental maps were performed (Fig. 4). Zr_0.5_(NiFeCuMnCo)_0.5_O_x_ was composed of small NPs (e.g., 10–30 nm), which aggregated together and resulted in abundant interstitial porosity. This feature was in agreement with the found N_2_ sorption isotherm of Zr_0.5_(NiFeCuMnCo)_0.5_O_x_ (S_*BET*_ = 84 m^2^ g^−1^). Moreover, the EDS elemental maps of individual metals showed that the five dopants were homogeneously distributed in the ZrO_2_ backbone, suggesting that five metals-doped ZrO_2_ solid solution was constructed. Meanwhile, the molar ratio of elements (Zr:Ni:Fe:Cu:Mn:Co) in Zr_0.5_(NiFeCuMnCo)_0.5_O_x_ material was confirmed to be 5.7:1:1.1:1.1:1.1:1.1 by inductively coupled plasma mass spectrometer (Supplementary Table 2), which matched well with the theoretical formula. These investigations encouraged us to explore its self-regeneration possibility during redox cycles.

### The exploration of self-regeneration process

To verify the exsolution and reintegration behavior, several exploratory experiments were carried out with high entropy Zr_0.5_(NiFeCuMnCo)_0.5_O_x_ as a model. The exsolution of metals was determined by the reducibility of transition metal oxides^48^, and it was closely related to co-segregation energy (transition metal accompanying with oxygen vacancies)^49^. The ZrO_2_ doped with low valence metals could contribute to produce oxygen vacancies^50^, which promoted the exsolution of metals^51^ under reductive atmosphere. Subsequently, the H_2_-TPR spectrum was tested to probe the reduction behavior of Zr_0.5_Co_0.5_O_x_, Zr_0.5_Ni_0.5_O_x_, Zr_0.5_Cu_0.5_O_x_, Zr_0.5_Fe_0.5_O_x_, Zr_0.5_Mn_0.5_O_x_, Zr_0.5_Mn_0.25_Cu_0.25_O_x_ (Supplementary Fig. 4) and pristine Zr_0.5_(NiFeCuMnCo)_0.5_O_x_ samples (Supplementary Fig. 5). As shown in Supplementary Fig. 4a, the peak at 256 °C resulted from the reduction of CuO to Cu^52^. For Zr_0.5_Mn_0.5_O_x_ sample (Supplementary Fig. 4b), the two reduction peaks were considered as MnO_2_ or Mn_2_O_3_ to Mn_3_O_4_ (318 °C), and Mn_3_O_4_ to MnO (420 °C)^53^. For Zr_0.5_Mn_0.25_Cu_0.25_O_x_ sample (Supplementary Fig. 4c), the reduction peaks at 269 °C and 325 °C were attributed to the reduction of the CuO to Cu^0^ and manganese oxides to MnO, respectively^54^. All three types of nickel oxide (363, 467, 570 °C) were reduced to the Ni^0^ (Supplementary Fig. 4d)^55^. For Zr_0.5_Fe_0.5_O_x_ sample (Supplementary Fig. 4e), the TPR profile showed two distinct reduction peaks. The peak centered at 401 °C was assigned to the reduction of Fe_2_O_3_ to Fe_3_O_4_ and the peak at 566 °C was ascribed to the reduction of Fe_3_O_4_ to Fe^0^ ^56^. For cobalt-doped sample (Supplementary Fig. 4f), the reduction peaks around 295 °C and 408 °C were attributed to the transitions from Co^3+^ to Co^2+^ and Co^2+^ to Co^0^ ^57^. Indeed, the addition of CuO could decrease the reduction temperature (Supplementary Fig. 4a, b, c)^54^. Interestingly, only one strong reduction peak was observed at ~279 °C for Zr_0.5_(NiFeCuMnCo)_0.5_O_x_ sample (Supplementary Fig. 5). Then, with CuO as the standard reference, the H_2_ consumption of Zr_0.5_(NiFeCuMnCo)_0.5_O_1.1+y_ sample (1.1 ascribes to unreducible ZrO_2_^58^, and MnO^49^) can reach 3.63 mmol g^−1^. Then, the y value is calculated to be 0.36^59^. In addition, the y values of other samples were provided (Supplementary Table 3). Based on this result, the Zr_0.5_(NiFeCuMnCo)_0.5_O_1.46_ formula suggests that significant amount of oxygen vacancies were created. Hence, these preliminary studies encourage further research about the exsolution process.

**Fig. 5:**
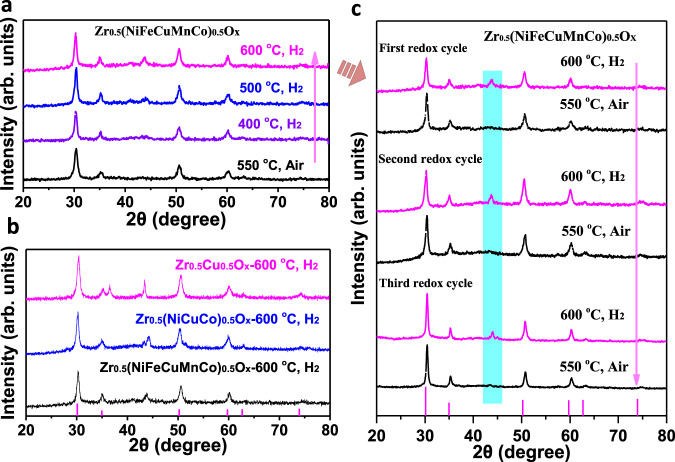
The XRD patterns of doped ZrO_x_ samples during redox processes. **a** The P-XRD patterns of Zr_0.5_(NiFeCuMnCo)_0.5_O_x_ treated at 10% H_2_ balance with N_2_ at appoint temperature (400, 500, 600 °C) for 2 h. **b** The P-XRD patterns of Zr_0.5_Cu_0.5_O_x_, Zr_0.5_(CuCoNi)_0.5_O_x_, and Zr_0.5_(NiFeCuMnCo)_0.5_O_x_ treated at 10% H_2_ balance with N_2_ at 600 °C for 2 h. **c** the P-XRD patterns of phase evolution in multi cycles between reductive and oxidative conditions. arb. units: arbitrary unit.

Subsequently, the pristine high entropy Zr_0.5_(NiFeCuMnCo)_0.5_O_x_ was treated in reduction condition (H_2_) at appointed temperatures for 2 h. As shown in Fig. 5a, the early stage of phase transformation was not clear at 400 °C. When the reduction temperature raised to 500–600 °C, the diffraction pattern of the exsolution phase showed a broad peak at 44 °C, which could be assigned to the reflection of transition metals (111). At the same time, typical peaks for cubic ZrO_2_ were still preserved during H_2_ treatment. In sharp contrast, the exsolution diffraction peaks of as-prepared binary Zr_0.5_Cu_0.5_O_x_ were sharp, revealing the excessive growth of Cu particles (average particle size by Reitveld refinement analysis: 37.7 nm)^60^ (Fig. 5b, Supplementary Fig. 6). Meanwhile, the exsolution peaks of quaternary Zr_0.5_(NiCuCo)_0.5_O_x_ presented two different phases (Fig. 5b). The multi redox behavior of Zr_0.5_(NiFeCuMnCo)_0.5_O_x_ was detailedly studied in the following (Fig. 5c). Therefore, high-entropy Zr_0.5_(NiFeCuMnCo)_0.5_O_x_ as a host matrix could somewhat suppress the growth or agglomeration of exsolved metal NPs even under harsh reductive conditions (e.g., 600 °C), which stood on the surface of parent high entropy ZrO_2_ NPs.

**Fig. 6:**
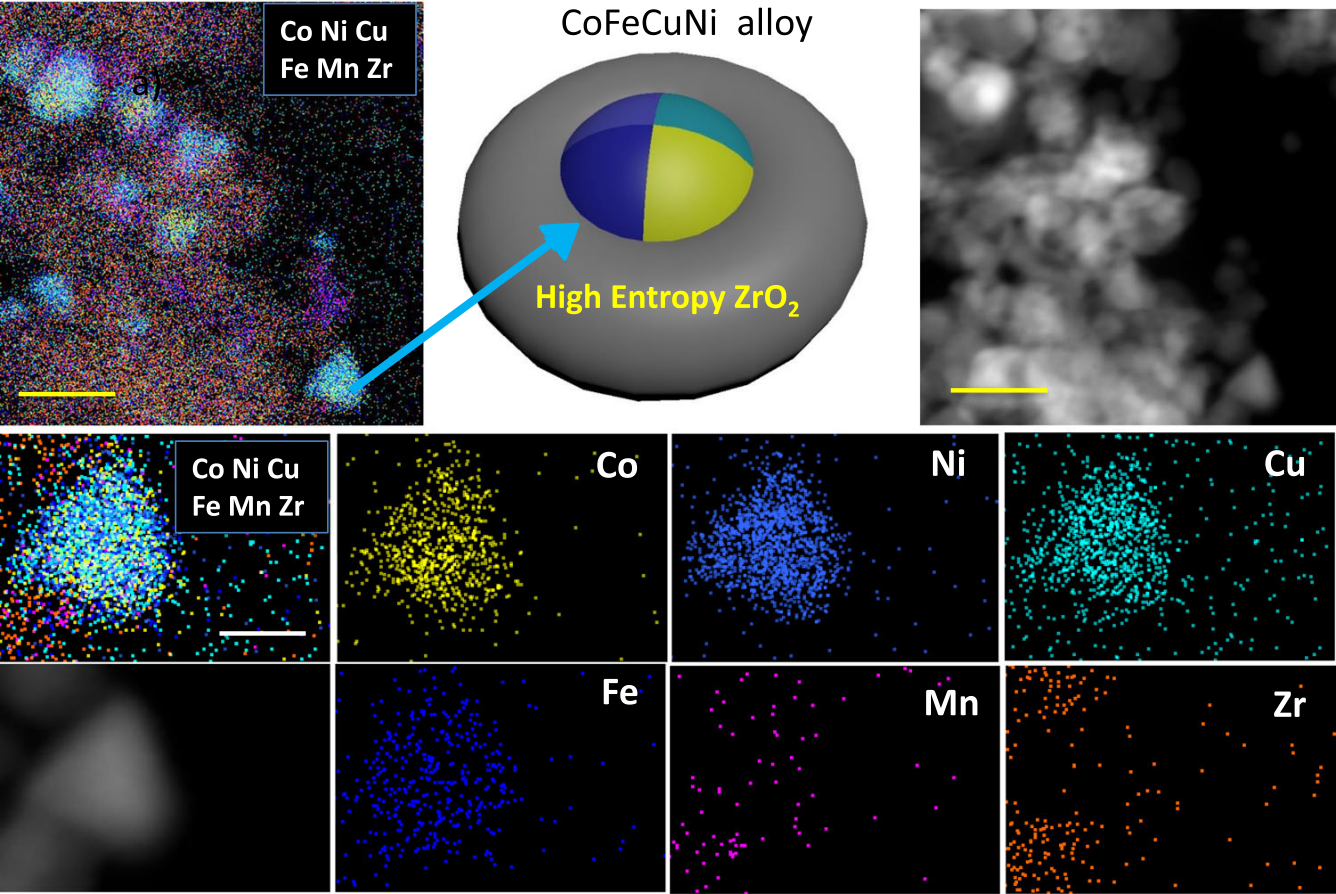
The characterization of H_2_-reduced Zr_0.5_(NiFeCuMnCo)_0.5_O_x_. STEM-HAADF images and EDS elemental maps of Zr_0.5_(NiFeCuMnCo)_0.5_O_x_ (treated at 400 °C, H_2_). Yellow scale bar, 30 nm. White scale bar, 10 nm.

Then, the Zr_0.5_(NiFeCuMnCo)_0.5_O_x_ (treated at 400 °C, H_2_) was investigated by STEM and elemental mapping analysis. Firstly, STEM was employed to provide insights into the exsolved NPs in Zr_0.5_(NiFeCuMnCo)_0.5_O_x_ under reducing atmosphere. As shown in Supplementary Fig. 7, reductive H_2_ treatment induced the exsolution of grains growing on the surface of parent high entropy ZrO_2_ NPs. Meanwhile, elemental mapping analysis exhibited aggregation of some elements (Supplementary Fig. 7b). To further confirm the exsolved metal species, the high resolution elemental mapping images at the edge of the sample revealed the formation of the CoFeCuNi alloy NPs (Fig. 6). To further study clearly the metal exsolution, the O elemental mapping was added (Supplementary Fig. 8). A typical particle on the edge was highlighted by yellow cycle. It showed a relatively low O content, close to the background. This result somewhat demonstrated the formation of the metallic CoFeCuNi particle.

**Fig. 7:**
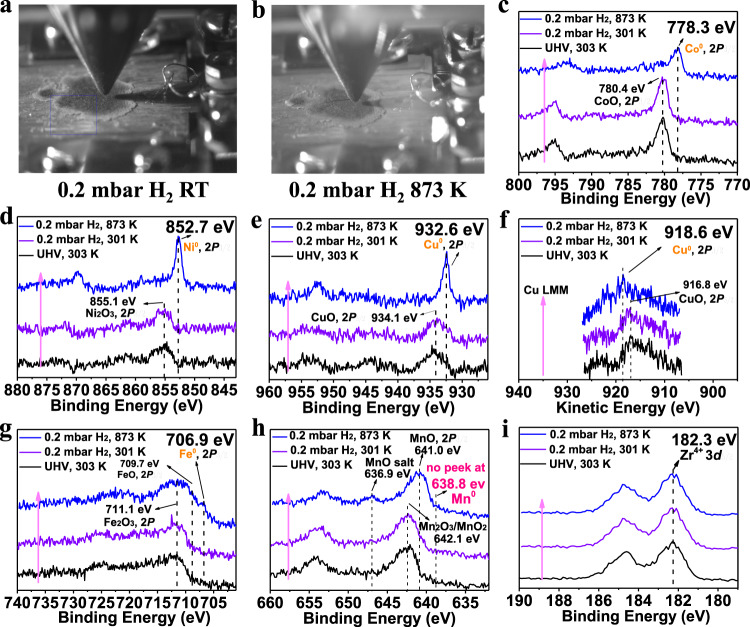
In situ *XPS* measurements of Zr_0.5_(NiFeCuMnCo)_0.5_O_x_. The sample was treated with X-ray exposure under UHV (traditionally ultrahigh vacuum) conditions (0.2 mbar H_2_). **a** Photograph of Zr_0.5_(NiFeCuMnCo)_0.5_O_x_ under NAPXPS test at room temperature. **b** Photograph of Zr_0.5_(NiFeCuMnCo)_0.5_O_x_ under NAPXPS test at 873 K. **c** Co 2p region. **d** Ni 2p region. **e**, **f** Cu 2p region. **g** Fe 2p region. **h** Mn 2p region. **i** Zr 3d region.

To further understand current observation, in situ high-resolution *XPS* (Fig. 7a, b) was carried out to investigate the surface state of the Zr_0.5_(NiFeCuMnCo)_0.5_O_x_ during H_2_ treatment from RT to 873 K. As shown in Fig. 7c, the peak centered at 778.3 eV was assigned to the characteristic Co 2*P*_3/2_ peak of Co^0^ ^61^. Meanwhile, the sharp peak at 852.7 eV resulted from surface Ni^0^ (Fig. 7d)^61^. Since the Binding Energy of Cu^+^ generally overlaps with Cu^0^ in the Cu 2 *P* core level (Fig. 7e, peak at 932.6 eV), the X-ray induced Auger electron spectra in the kinetic energy region of 928–906 eV is provided in Fig. 7f. As shown in Fig. 7f, the kinetic energy at 918.6 eV could be contributed to Cu^0^ ^61^. A signal peak is located at 706.9 eV, which is ascribed to the Fe 2*P*_3/2_ metal (Fig. 7g)^61^. As expected, there is no peak for Mn^0^ at 638.8 eV^61^. Meanwhile, MnO 2*P*_3/2_ (at 641.0 eV) peaks show their characteristic satellite peak at 636.9 eV (Fig. 7h)^62^. Indeed, manganic oxide is more difficult to be reduced to Mn^0^ ^63^. Under H_2_ treatment, several transition metal ions (Co, Ni, Cu, Fe) seem to in situ migrate and separate from the bulk of high entropy Zr_0.5_(NiFeCuMnCo)_0.5_O_x_, while the original cubic ZrO_2_ matrix was well maintained (Fig. 5a). The H_2_-induced formation of exsolved CoNiCuFe NPs is different with the Cantor alloy (FeCrMnNiCo) by melting process^64^ and those CoNiCuFe NPs could not be described as high entropy alloys^65^. As illustrated in Supplementary Fig. 5, only one strong reduction peak was observed at ~279 °C, which could be assigned to the produce of the CoFeCuNi alloy. Based on these results, the exsolution phenomenon of alloys occurred under reductive atmosphere, which was just the first step of self-regenerative process.

**Fig. 8:**
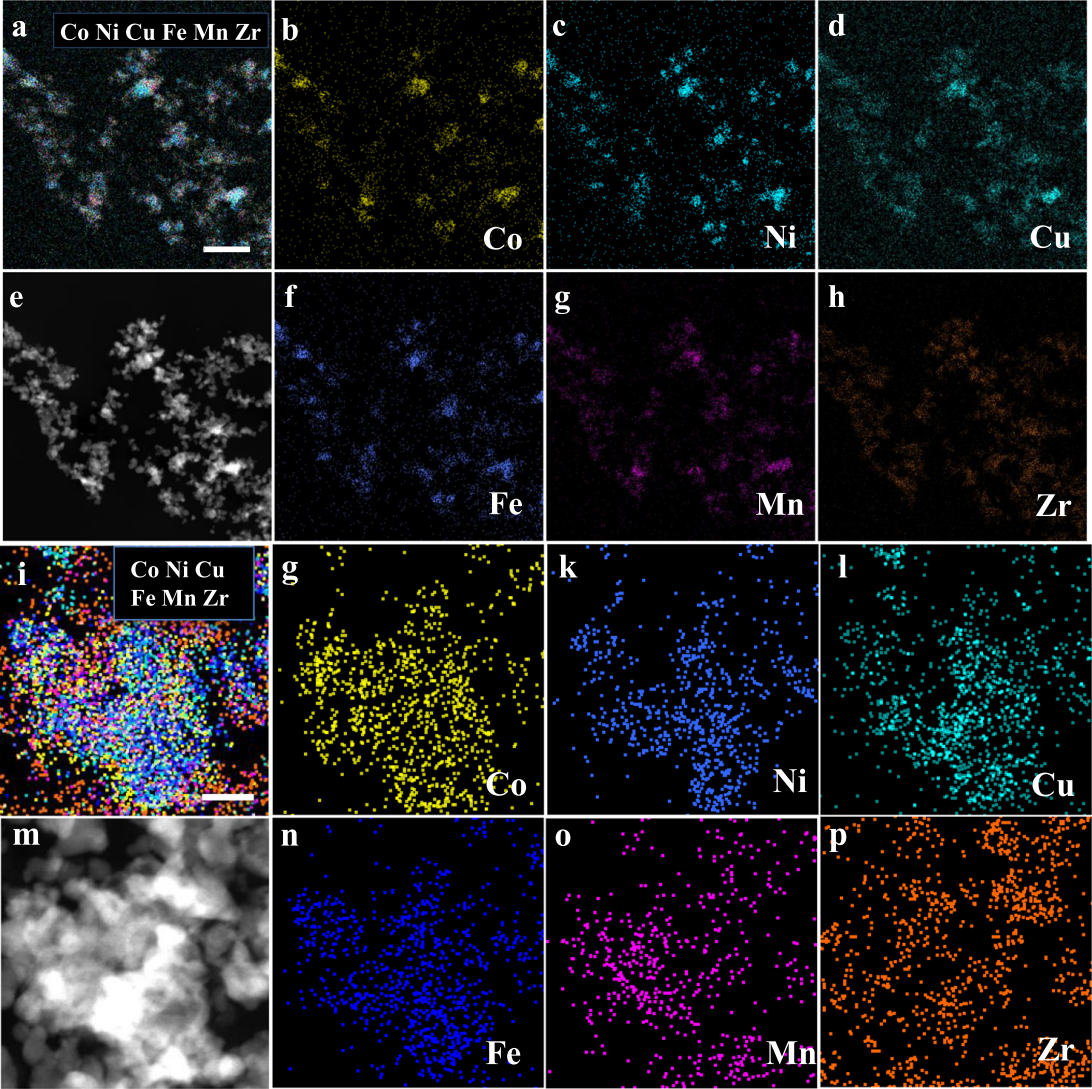
The characterization of Zr_0.5_(NiFeCuMnCo)_0.5_O_x_ after three redox cycles (600 °C H_2_/550 °C, Air). **a**–**h** STEM-HAADF image and EDS elemental maps of Zr_0.5_(NiFeCuMnCo)_0.5_O_x_ after three redox cycles. Scale bar, 200 nm. **i**–**p** STEM-HAADF image and EDS elemental maps of Zr_0.5_(NiFeCuMnCo)_0.5_O_x_ after three redox cycles. Scale bar, 50 nm.

To figure out the self-regeneration possibility of high entropy Zr_0.5_(NiFeCuMnCo)_0.5_O_x_, the dissolution process was subsequently explored. The reduced Zr_0.5_(NiFeCuMnCo)_0.5_O_x_ (treated at 600 °C, H_2_) was further exposed to oxidative environment (550 °C, air). After this cycle, the obvious XRD peaks for CoFeCuNi alloy phase disappeared (Fig. 5c). The interesting exsolution-reintegration behavior of the high entropy ZrO_2_ material was further proved by three redox cycles (Fig. 5c). The oxidized sample after three cycles was observed by STEM images. The elemental mapping images in different scale bars, such as 50 nm, 200 nm, 50 μm, have been taken. As shown in Fig. 8 and Supplementary Fig. 9, all five heterometal species were evenly distributed in ZrO_2_ matrix, which was the same as the pristine Zr_0.5_(NiFeCuMnCo)_0.5_O_x_ (Fig. 4). As expected, the entropy-added motivation may drive the reintegration of CoFeCuNi alloy NPs into the parent ZrO_2_ matrix in air, which contributed to the reversible self-regenerative process. All those results above suggested the feasibility of the high entropy ZrO_2_ as a host matrix for self-regenerative process.

### The catalytic performance of high entropy ZrO_2_ catalyst

**Fig. 9:**
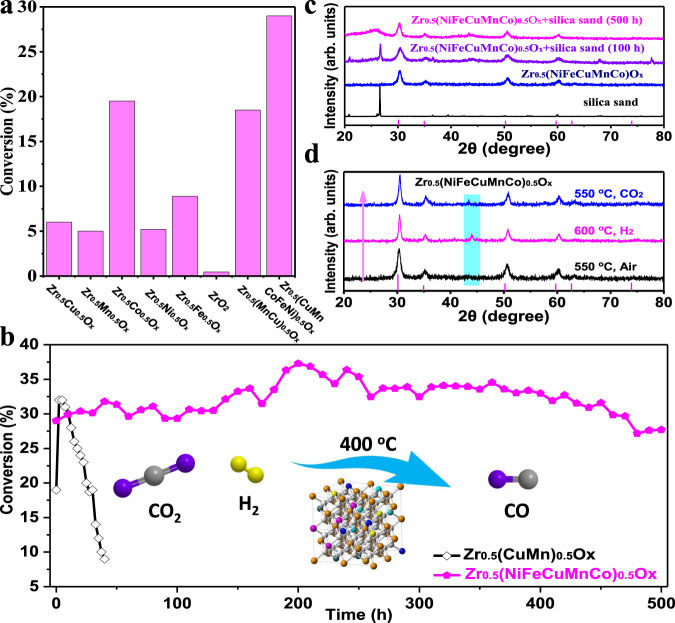
Catalytic performance of doped ZrO_2_ catalysts. **a** catalytic performance of Zr_0.5_Cu_0.5_O_x_, Zr_0.5_Mn_0.5_O_x_, Zr_0.5_Co_0.5_O_x_, Zr_0.5_Ni_0.5_O_x,_ Zr_0.5_Fe_0.5_O_x,_ Zr_0.5_(CuMn)_0.5_O_x,_ and Zr_0.5_(NiFeCuMnCo)_0.5_O_x_ in CO_2_ hydrogenation at 400 °C. **b** P-XRD patterns of reused Zr_0.5_(NiFeCuMnCo)_0.5_O_x_ samples after 100 h and 500 h on steam. **c** The P-XRD patterns of phase evolution between H_2_/CO_2_ conditions. **d** Thermal stability tests of Zr_0.5_(NiFeCuMnCo)_0.5_O_x_ and Zr_0.5_(CuMn)_0.5_O_x_ in CO_2_ hydrogenation. arb. units: arbitrary unit.

Then, the activity and thermal stability of high entropy Zr_0.5_(NiFeCuMnCo)_0.5_O_x_ were explored in high temperature catalysis (Fig. 9). CO_2_ hydrogenation (reverse water gas shift, RWGS), with an enthalpy ΔH of 41.3 kJ mol^−1^, is a typical endothermic reaction^66^. From the sight of both kinetics and thermodynamic, high temperature is more conducive to accelerating the reaction rate and moving the equilibrium in the direction of CO generation^67^. In other words, RWGS reaction is usually performed at high temperatures (e.g., 300–500 °C), whose catalysts are easy to be sintered^68^. In addition, a redox reaction mechanism of active sites was proposed to explain this process^69^. Therefore, RWGS reaction was applied as a model process to evaluate the high entropy ZrO_2_ catalyst.

Compared with binary and ternary doped ZrO_2_ catalysts (Zr_0.5_Cu_0.5_O_x_, Zr_0.5_Mn_0.5_O_x_, Zr_0.5_Co_0.5_O_x_, Zr_0.5_Ni_0.5_O_x,_ Zr_0.5_Fe_0.5_O_x,_ Zr_0.5_(CuMn)_0.5_O_x_) (Supplementary Fig. 10a–d), the Zr_0.5_(NiFeCuMnCo)_0.5_O_x_ afforded better catalytic activity during CO_2_ hydrogenation (300–400 °C). When the temperature reached 400 °C, the CO_2_ conversion was ~29% by Zr_0.5_(NiFeCuMnCo)_0.5_O_x_ and the selectivity for CO is over 90% (Fig. 9a, Supplementary Fig. 10e). Then, the long-term stability of Zr_0.5_(NiFeCuMnCo)_0.5_O_x_ during CO_2_ hydrogenation reaction was studied. No obvious loss of catalytic activity and selectivity was found during continuous CO_2_ hydrogenation for 500 h (Fig. 9b). In sharp contrast, severe deactivation by the ternary doped Zr_0.5_(MnCu)_0.5_O_x_ catalyst was observed in relatively short reaction time (40 h) (Fig. 9b). Meanwhile, the XRD pattern of spent Zr_0.5_(NiFeCuMnCo)_0.5_O_x_ catalyst showed little tendency towards segregation or sintering, further illustrating the advantage of high entropy (Zr_0.5_(NiFeCuMnCo)_0.5_O_x_) as the host structure (Fig. 9c). In addition, the dissolution of metal alloys somewhat underwent during CO_2_ atmosphere (600 °C), as suggested by the diminished XRD peaks for metallic species (Fig. 9d). Furthermore, control experiments were carried out for comparison. First, the 5%(NiFeCuCo)/ZrO_x_ and 10%(NiFeCuCo)/ZrO_x_ were synthesized by wet-impregnation method. The long-term stability of supported NiFeCuCo catalyst during CO_2_ hydrogenation reaction was studied at the same condition (Supplementary Fig. 11). Unfortunately, severe deactivation was observed in relatively short reaction time (1 h and 3 h). Compared with the high entropy Zr_0.5_(NiFeCuMnCo)_0.5_O_x_ material (>500 h), 5%(NiFeCuCo)/ZrO_x_ and 10%(NiFeCuCo)/ZrO_x_ exhibited poor stability. Based on those results, unique properties of the high entropy structure indeed promoted the thermal stability of doped ZrO_2_ catalysts.

## Discussion

In summary, a cubic high-entropy Zr_0.5_(NiFeCuMnCo)_0.5_O_x_ was designed and simply prepared. The contribution of high entropy for the sintering-resistance ability of supported transition metals was figured out, while severe particle growth occurred on Zr_0.5_Cu_0.5_O_x_ and Zr_0.5_(CuCoNi)_0.5_O_x_ treated in H_2_. Meanwhile, the reversible exsolution-dissolution of fine CoFeCuNi alloys on high entropy Zr_0.5_(NiFeCuMnCo)_0.5_O_x_ was observed in H_2_/Air treatments. Those results somewhat validate that entropy-driving force may contribute to self-regenerative process and sintering-resistance performance of supported transition metals. During high temperature catalysis (400 °C), no obvious loss of catalytic activity and little tendency towards segregation were found during continuous CO_2_ hydrogenation for 500 h, once again arguing for the excellent sintering-resistance ability of high entropy catalysts.

The high entropy oxide was invented in 2015^29^ and related catalysis has been unveiled in around 2018^70^, and their unexpected talent for heterogeneous catalysis is a young topic^63^, yet full of promise. The idea of considering entropic contribution for stabilized catalyst is just the beginning, and more unique materials for heterogeneous catalysis would be inspired in the near future.

## Methods

Chemicals involved in the manuscript are used directly without purification. ZrCl_4_ (>99.9%, Aladdin), NiCl_2_ (>99%, Adamas), FeCl_3_ (>99%, Adamas), CuCl_2_·2H_2_O ( > 99%, Greagent), MnCl_2_ (>99%, Greagent), CoCl_2_·6H_2_O ( > 98%, Adamas), NaOH (>96%, Greagent), NaCl (>99.5%, Greagent).

The preparation of entropy-stabilized, highly doped microstructure Zr_0.5_(NiFeCuMnCo)_0.5_O_x_ is as follows. ZrCl_4_ (2.5 mmol, 5 eq.), NiCl_2_ (0.5 mmol, 1 eq.), FeCl_3_ (0.5 mmol, 1 eq.), CuCl_2_•2H_2_O (0.5 mmol, 1 eq.), MnCl_2_ (0.5 mmol, 1 eq.), CoCl_2_•6H_2_O (0.5 mmol, 1 eq.), and NaCl (2 g) were whisked together in a 50 mL ZrO_2_-milling equipment with three ball-bearings (1× diameter 1.2 cm, 2× diameter 0.8 cm). The mixtures were ball milled for 1 h. Then the NaOH (15.5 mmol) was added into the system and the mixtures were ball milled for another 1 h. Calcination of the as-synthesized powder was performed into a muffle oven in air at 400 °C, 500 °C, 550 °C, 600 °C for 3 h (2 K min^−1^ to appointed temperature) and then cooled down to room temperature. The powder was washed three times by deionized water, and then washed with ethanol. Then as-made samples (Zr_0.5_(NiFeCuMnCo)_0.5_O_x_) were put into a vacuum drying oven and dried at 70 °C for 12 h. The synthesis of other doped ZrO_2_ materials for comparative experiments can be found in Supplementary Methods section. The obtained samples were referred to as Zr_0.5_Cu_0.5_O_x_, Zr_0.5_Mn_0.5_O_x_, Zr_0.5_Co_0.5_O_x_, Zr_0.5_Ni_0.5_O_x,_ Zr_0.5_Fe_0.5_O_x,_ Zr_0.5_(CuMn)_0.5_O_x_, Zr_0.5_(NiFeCuMnCo)_0.5_O_x_.

The powder X-ray diffraction (D8 Advance, Bruker) operated at 40 kV and 40 mA and the pattern was recorded in the range of 20–80°. The material was characterized by N_2_ adsorption isotherms (Tristar II 3020 S1N1878) at 77 K. NAPXPS measurements were carried out on an SPECS system equipped with a differentially pumped Phoibos hemispherical electron energy analyzer using monochromatic Al Kα radiation (1486.6 eV). The in situ characterization was performed in the 873 K 0.2 mbar H_2_ reduction condition. The heating rate was 5 K min^−1^ from 301 K to 873 K. HAADF-STEM (Talos F200X G2, FEI). Leica EM TXP (Mingrui GC2060, China). Inductive Coupled Plasma Emission Spectrometer (iCAP7600, made by Thermo., USA).

The as-made catalysts (75 or 50 mg) were used for the H_2_-TPR experiments (Micromeritics Autochem II 2920). The material was pretreated in Ar (30 mL min^−1^) at 300 °C for 60 min and then cooled to the room temperature. Then, the 5% H_2_ in Ar (30 mL min^−1^) was switched on in a heating rate of 10 °C min^−1^. Finally, the H_2_ consumption of the as-made materials was monitored by a TCD detector.

The RWGS reaction worked at atmospheric pressure in a fixed-bed quartz reactor. Firstly, 30 mg catalyst and 100 mg quartz sand were mixed together well. Then, the catalyst with quartz was exposed to a stream of feed gas (H_2_:CO_2_ = 3:1, 10 mL min^−1^). When the catalyst reached the appointed temperature, the gaseous products were analyzed by gas chromatograph with a TCD and FID detector.

Detailed procedures are in the Supplementary Methods section.

## Supplementary information

Supplementary Information

## Data Availability

The authors declare data supporting the findings of this study are available within the paper and its Supplementary Information. All data are available from the authors on reasonable request.
