# Supplementary material for: Self-regeneration of supported transition metals by a high entropy-driven principle
Source: Nat Commun. 2021 Oct 11;12:5917. doi: 10.1038/s41467-021-26160-8 (PMC8505510; doi:10.1038/s41467-021-26160-8)
Supplement: Supplementary file 1 — Supplementary Information [file 41467_2021_26160_MOESM1_ESM.pdf]

Supporting Information for Self-Regeneration of Supported Transition Metals by a High Entropy-Driven Principle

Supplementary Information includes:

Supplementary Methods

Supplementary Figures

Supplementary Tables

Supplementary Methods

$Zr_{0.5}(NiFeCuMnCo)_{0.5}O_x$: $ZrCl_4$ (2.5 mmol, 5 eq.), $NiCl_2$ (0.5 mmol, 1 eq.), $FeCl_3$ (0.5 mmol, 1 eq.), $CuCl_2$•$2H_2O$ (0.5 mmol, 1 eq.), $MnCl_2$ (0.5 mmol, 1 eq.), $CoCl_2$• $6H_2O$ (0.5 mmol, 1 eq.), and NaCl (2 g) were whisked together in a 50 mL ZrO_2-milling equipment with three ball-bearings (1 X diameter 1.2 cm, 2 X diameter 0.8 cm). The mixtures were ball milled for 1 h. Then the NaOH (15.5 mmol) was added into the system and the mixtures were ball milled for another 1 h. Calcination of the as-synthesized powder was performed into a muffle oven in air at 400 ^oC, 500 ^oC, 550 ^oC, 600 ^oC for 3 h (2 K min^{-1} to appointed temperature) and then cooled down to room temperature. The powder was washed three times by deionized water, and then washed with ethanol. Then, it was put into a vacuum drying oven and dried at 70 ℃ for 12 hours.

$Zr_{0.5}Cu_{0.5}O_x$: $ZrCl_4$ (2.5 mmol, 1 eq.), $CuCl_2$•$2H_2O$ (2.5 mmol, 1 eq.) and NaCl (2.2 g) were whisked together in a 50 mL ZrO_2-milling equipment with three ball-bearings (1 X diameter 1.2 cm, 2 X diameter 0.8 cm). The mixtures were ball milled for 1 h. Then the NaOH (15.5 mmol) was added into the system and the mixtures were ball milled for another 1 h. Calcination of the as-synthesized powder was performed into a muffle oven in air at 550 ^oC for 3 h (2 K min^{-1} to appointed temperature) and then cooled down to room temperature. The powder was washed three times by deionized water, and then washed with ethanol. Then, it was put into a vacuum drying oven and dried at 70 ℃ for 12 hours.

$Zr_{0.5}Mn_{0.5}O_x$: $ZrCl_4$ (2.5 mmol, 1 eq.), $MnCl_2$ (2.5 mmol, 1 eq.) and NaCl (2 g) were

whisked together in a 50 mL ZrO_2-milling equipment with three ball-bearings (1 X diameter 1.2 cm, 2 X diameter 0.8 cm). The mixtures were ball milled for 1 h. Then the NaOH (15.5 mmol) was added into the system and the mixtures were ball milled for another 1 h. Calcination of the as-synthesized powder was performed into a muffle oven in air at 550 ^{o}C for 3 h (2 K min^{-1} to appointed temperature) and then cooled down to room temperature. The powder was washed three times by deionized water, and then washed with ethanol. Then, it was put into a vacuum drying oven and dried at 70 ^{o}C for 12 hours.

$Zr_{0.5}Co_{0.5}O_x$: $ZrCl_4$ (2.5 mmol, 1 eq.), $CoCl_2$•$6H_2O$ (2.5 mmol, 1 eq.) and NaCl (2.4 g) were whisked together in a 50 mL ZrO_2-milling equipment with three ball-bearings (1 X diameter 1.2 cm, 2 X diameter 0.8 cm). The mixtures were ball milled for 1 h. Then the NaOH (15.5 mmol) was added into the system and the mixtures were ball milled for another 1 h. Calcination of the as-synthesized powder was performed into a muffle oven in air at 550 ^{o}C for 3 h (2 K min^{-1} to appointed temperature) and then cooled down to room temperature. The powder was washed three times by deionized water, and then washed with ethanol. Then, it was put into a vacuum drying oven and dried at 70 ^{o}C for 12 hours.

$Zr_{0.5}Fe_{0.5}O_x$: $ZrCl_4$ (2.5 mmol, 1 eq.), $FeCl_3$ (2.5 mmol, 1 eq.) and NaCl (2.3 g) were whisked together in a 50 mL ZrO_2-milling equipment with three ball-bearings (1 X diameter 1.2 cm, 2 X diameter 0.8 cm). The mixtures were ball milled for 1 h. Then the NaOH (18 mmol) was added into the system and the mixtures were ball milled for another 1 h. Calcination of the as-synthesized powder was performed into a muffle

oven in air at 550 ^oC for 3 h (2 K min^{-1} to appointed temperature) and then cooled down to room temperature. The powder was washed three times by deionized water, and then washed with ethanol. Then, it was put into a vacuum drying oven and dried at 70 ^oC for 12 hours.

$Zr_{0.5}Ni_{0.5}O_x$: $ZrCl_4$ (2.5 mmol, 1 eq.), $NiCl_2$ (2.5 mmol, 1 eq.) and NaCl (2 g) were whisked together in a 50 mL ZrO_2-milling equipment with three ball-bearings (1 X diameter 1.2 cm, 2 X diameter 0.8 cm). The mixtures were ball milled for 1 h. Then the NaOH (15.5 mmol) was added into the system and the mixtures were ball milled for another 1 h. Calcination of the as-synthesized powder was performed into a muffle oven in air at 550 ^oC for 3 h (2 K min^{-1} to appointed temperature) and then cooled down to room temperature. The powder was washed three times by deionized water, and then washed with ethanol. Then, it was put into a vacuum drying oven and dried at 70 ^oC for 12 hours.

$Zr_{0.5}(CuMn)_{0.5}O_x$: $ZrCl_4$ (2.5 mmol, 1 eq.), $CuCl_2 \cdot 2H_2O$ (1.25 mmol, 0.5 eq.) $MnCl_2$ (1.25 mmol, 0.5 eq.) and NaCl (2.1 g) were whisked together in a 50 mL ZrO_2-milling equipment with three ball-bearings (1 X diameter 1.2 cm, 2 X diameter 0.8 cm). The mixtures were ball milled for 1 h. Then the NaOH (15.5 mmol) was added into the system and the mixtures were ball milled for another 1 h. Calcination of the as-synthesized powder was performed into a muffle oven in air at 550 ^oC for 3 h (2 K min^{-1} to appointed temperature) and then cooled down to room temperature. The powder was washed three times by deionized water, and then washed with ethanol. Then, it was put into a vacuum drying oven and dried at 70 ^oC for 12 hours.

Supplementary Figures

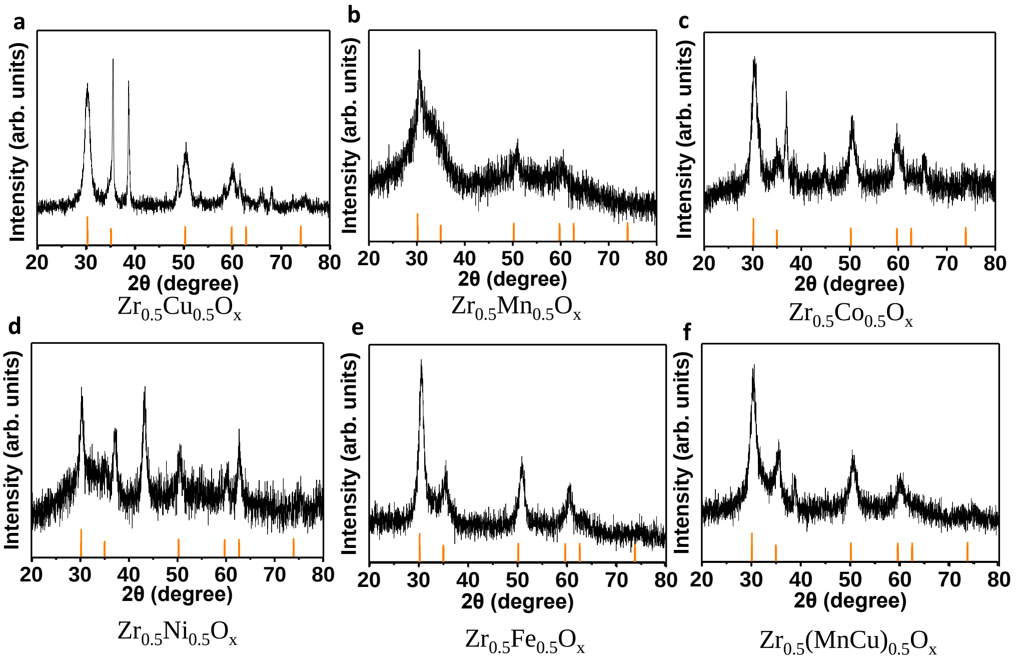

Supplementary Figure 1. **The PXRD patterns.** a $Zr_{0.5}Cu_{0.5}O_x$. b $Zr_{0.5}Mn_{0.5}O_x$. c $Zr_{0.5}Co_{0.5}O_x$. d $Zr_{0.5}Ni_{0.5}O_x$. e $Zr_{0.5}Fe_{0.5}O_x$. f $Zr_{0.5}(MnCu)_{0.5}O_x$. arb. units: arbitrary unit.

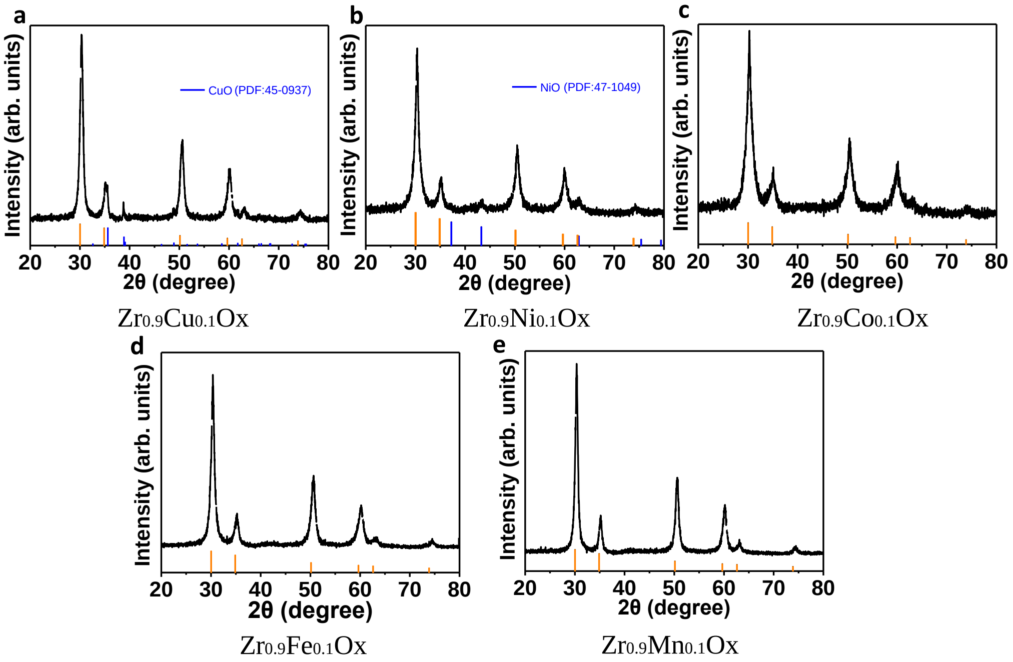

Supplementary Figure 2. The PXRD patterns. a $Zr_{0.9}Cu_{0.1}O_x$. b $Zr_{0.9}Ni_{0.1}O_x$. c $Zr_{0.9}Co_{0.1}O_x$. d $Zr_{0.9}Fe_{0.1}O_x$. e $Zr_{0.9}Mn_{0.1}O_x$. arb. units: arbitrary unit.

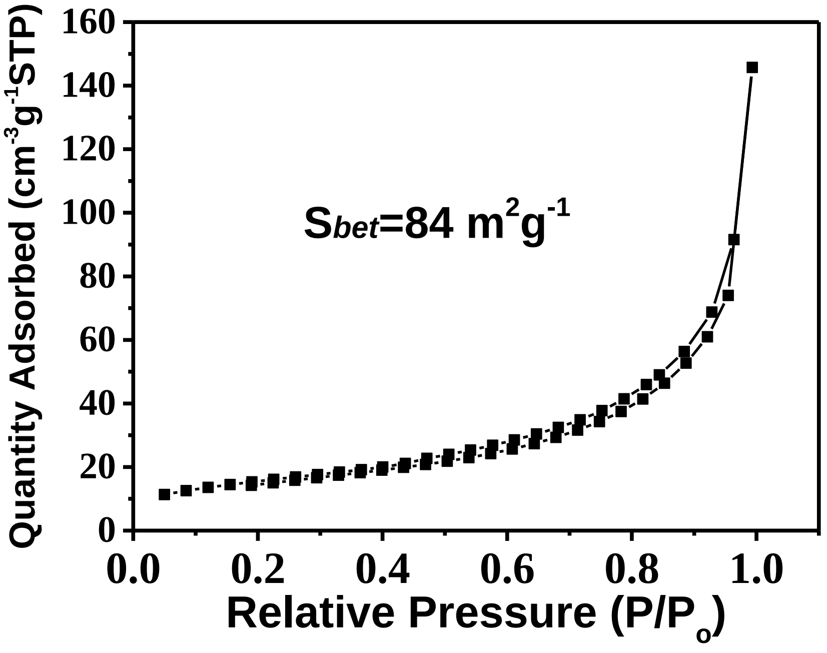

Supplementary Figure 3. The nitrogen sorption isotherm curves (77 K) of $Zr_{0.5}(NiFeCuMnCo)_{0.5}O_x$.

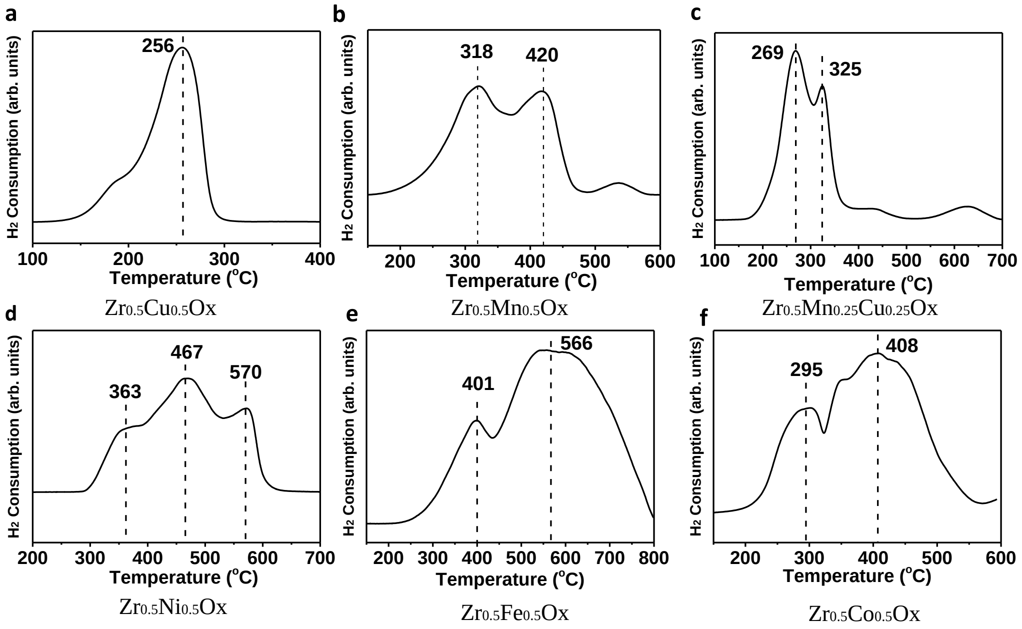

Supplementary Figure 4. **The H_2-TPR spectrum.** a $Zr_{0.5}Cu_{0.5}O_x$. b $Zr_{0.5}Mn_{0.5}O_x$. c $Zr_{0.5}Mn_{0.25}Cu_{0.25}O_x$. d $Zr_{0.5}Ni_{0.5}O_x$. e $Zr_{0.5}Fe_{0.5}O_x$. f $Zr_{0.5}Co_{0.5}O_x$. arb. units: arbitrary unit.

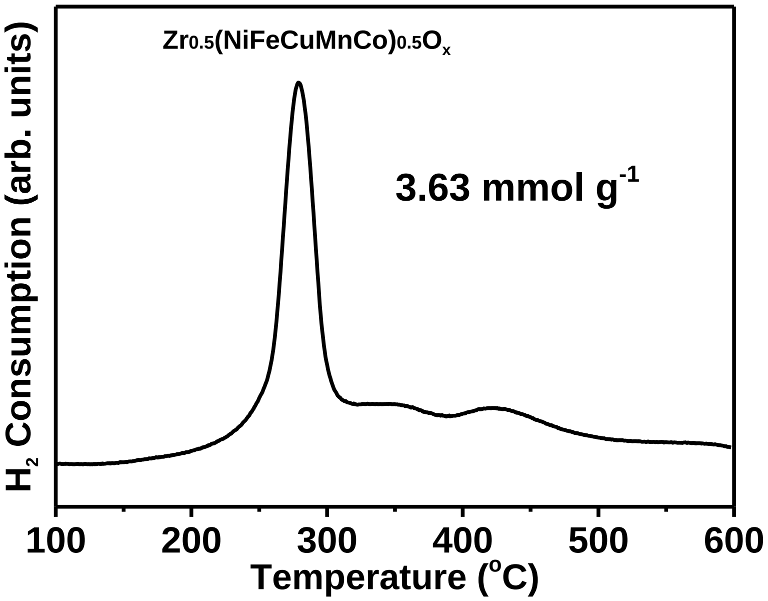

Supplementary Figure 5. **The H_2-TPR spectrum of $Zr_{0.5}(NiFeCuMnCo)_{0.5}O_x$.**

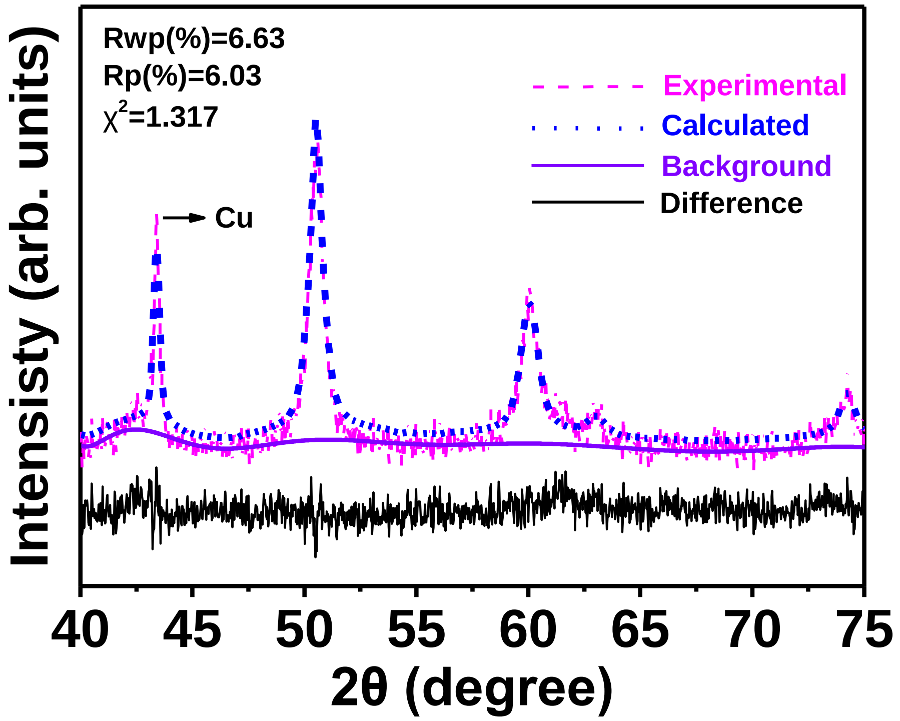

Supplementary Figure 6. P-XRD patterns together with Rietveld fits of the $Zr_{0.5}Cu_{0.5}O_X$ sample treated at 10% H_2 balance with N_2 at 600 ^oC for 2 h. The Cu particle size of reduced $Zr_{0.5}Cu_{0.5}O_x$ was calculated by Reitveld refinement analysis (K=1, λ=0.154056 nm). arb. units: arbitrary unit.

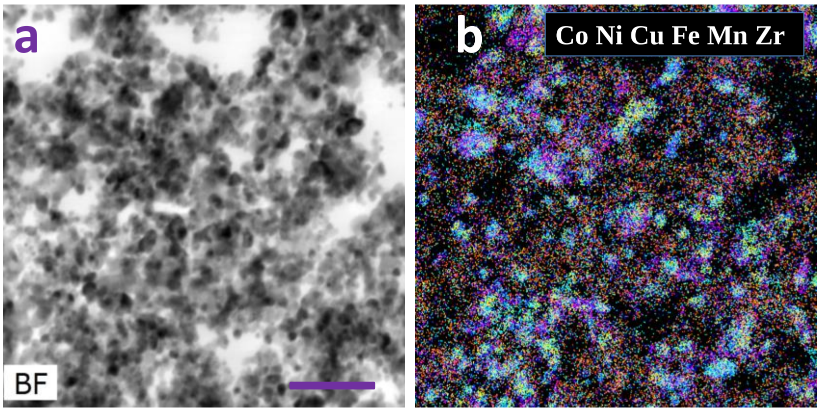

Supplementary Figure 7. The characterization of reduced $Zr_{0.5}(NiFeCuMnCo)_{0.5}O_x$. a TEM of reduced $Zr_{0.5}(NiFeCuMnCo)_{0.5}O_x$, b the EDS elemental maps of reduced $Zr_{0.5}(NiFeCuMnCo)_{0.5}O_x$; Scale bar, 100 nm.

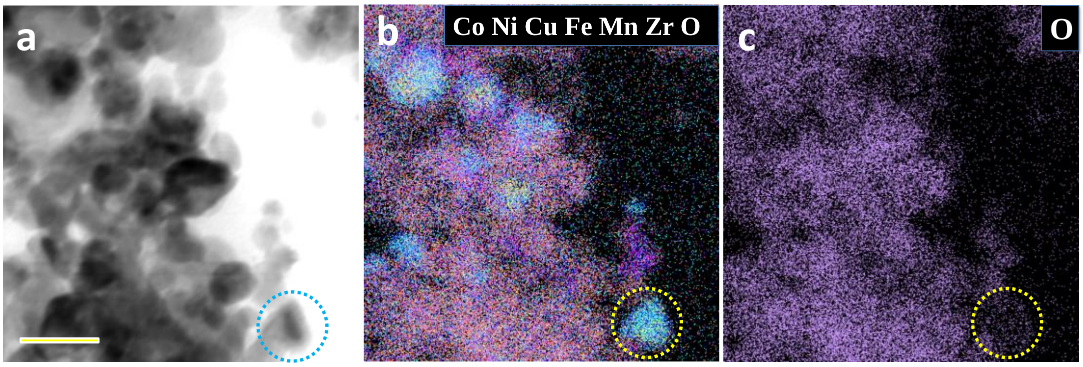

Supplementary Figure 8. The characterization of reduced $Zr_{0.5}(NiFeCuMnCo)_{0.5}O_x$. a TEM of reduced $Zr_{0.5}(NiFeCuMnCo)_{0.5}O_x$. b the EDS elemental maps of reduced $Zr_{0.5}(NiFeCuMnCo)_{0.5}O_x$, c the O elemental map of reduced $Zr_{0.5}(NiFeCuMnCo)_{0.5}O_x$; Scale bar, 30 nm.

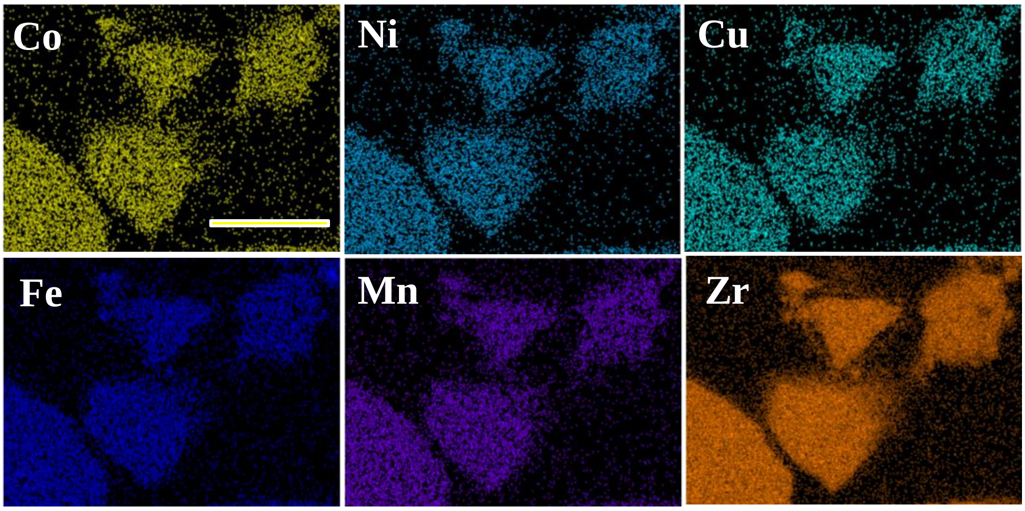

Supplementary Figure 9. EDS elemental maps of $Zr_{0.5}(NiFeCuMnCo)_{0.5}O_x$ after three redox cycles (600 ^oC H_2 / 550 ^oC Air). Scale bar, 50 μm. SEM-Mapping image: the sample was processed by Leica EM TXP.

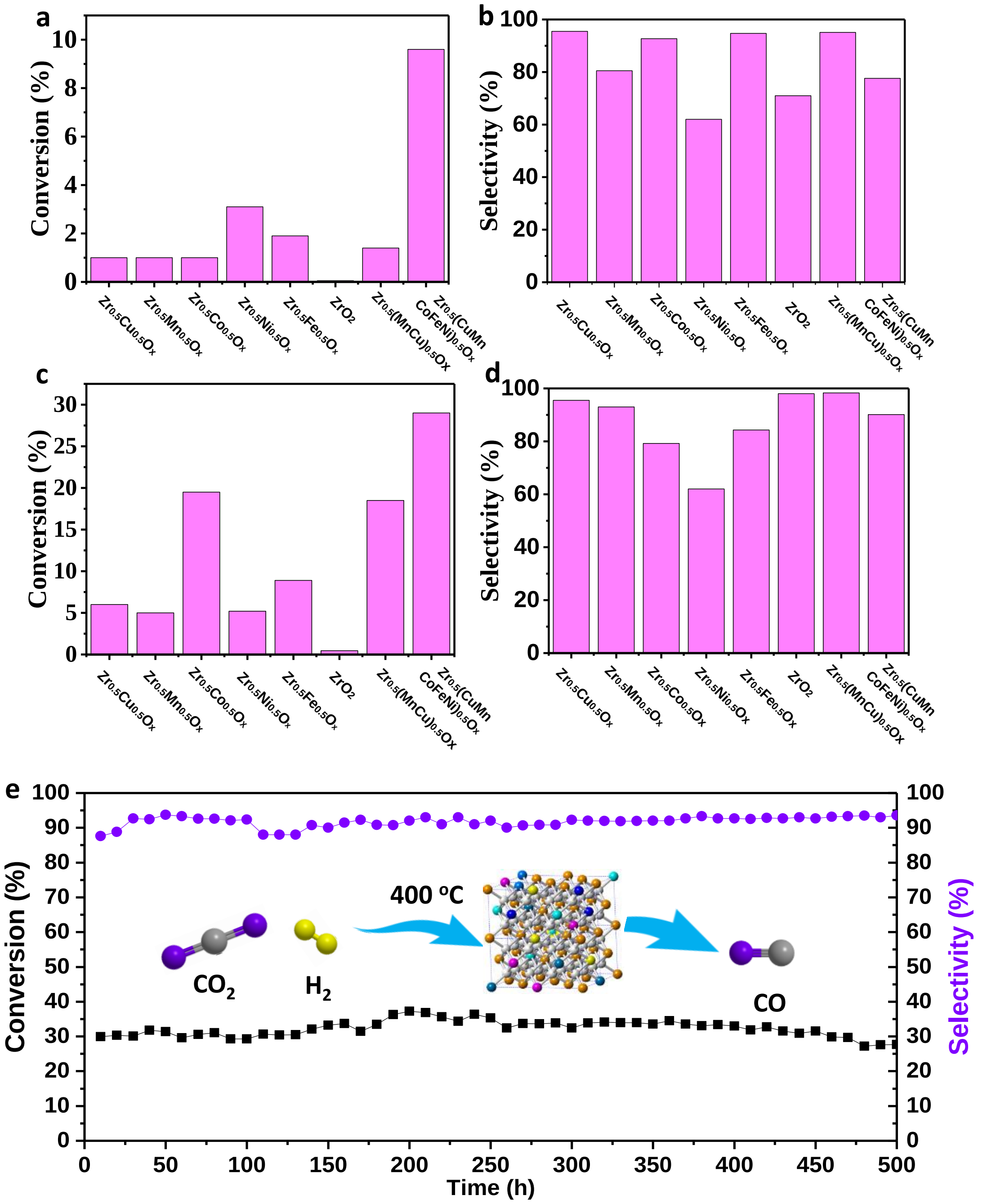

Supplementary Figure 10. Catalytic performance of doped ZrO_2 catalysts. a CO_2 conversion by $Zr_{0.5}Cu_{0.5}O_x$, $Zr_{0.5}Mn_{0.5}O_x$, $Zr_{0.5}Co_{0.5}O_x$, $Zr_{0.5}Ni_{0.5}O_x$, $Zr_{0.5}Fe_{0.5}O_x$, $Zr_{0.5}(MnCu)_{0.5}O_x$, and $Zr_{0.5}(NiFeCuMnCo)_{0.5}O_x$ in RWGS reaction at 300 ^oC. b CO selectivity by $Zr_{0.5}Cu_{0.5}O_x$, $Zr_{0.5}Mn_{0.5}O_x$, $Zr_{0.5}Co_{0.5}O_x$, $Zr_{0.5}Ni_{0.5}O_x$, $Zr_{0.5}Fe_{0.5}O_x$, $Zr_{0.5}(MnCu)_{0.5}O_x$, and $Zr_{0.5}(NiFeCuMnCo)_{0.5}O_x$ in RWGS reaction at 300 ^oC. c CO_2 conversion by $Zr_{0.5}Cu_{0.5}O_x$, $Zr_{0.5}Mn_{0.5}O_x$, $Zr_{0.5}Co_{0.5}O_x$, $Zr_{0.5}Ni_{0.5}O_x$, $Zr_{0.5}Fe_{0.5}O_x$, $Zr_{0.5}(MnCu)_{0.5}O_x$, and $Zr_{0.5}(NiFeCuMnCo)_{0.5}O_x$ in RWGS reaction at 400 ^oC. d CO selectivity by $Zr_{0.5}Cu_{0.5}O_x$, $Zr_{0.5}Mn_{0.5}O_x$, $Zr_{0.5}Co_{0.5}O_x$, $Zr_{0.5}Ni_{0.5}O_x$, $Zr_{0.5}Fe_{0.5}O_x$, $Zr_{0.5}(MnCu)_{0.5}O_x$, and $Zr_{0.5}(NiFeCuMnCo)_{0.5}O_x$ in RWGS reaction at 400 ^oC. e Thermal stability test in RWGS reaction at 400 ^oC.

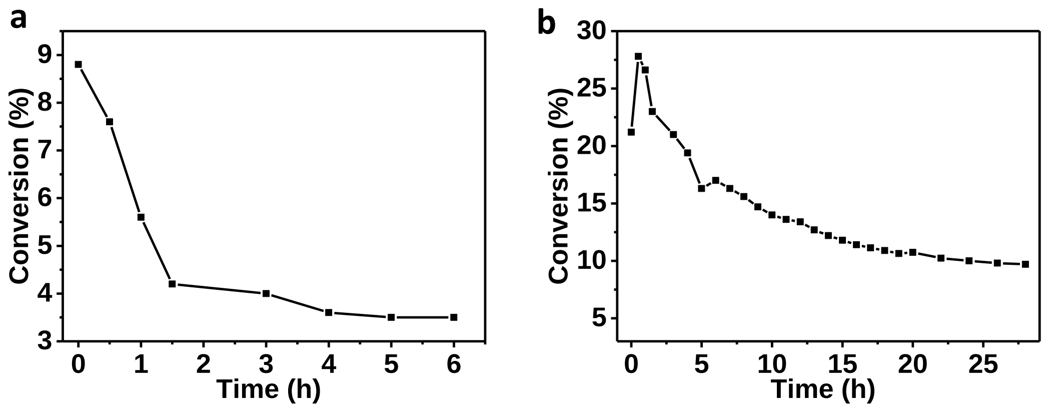

Supplementary Figure 11. Catalytic performance of control supported ZrO_2 catalysts by impregnation method. Thermal stability tests of a) 5%(NiFeCuCo)/ZrOx and b) 10%(NiFeCuCo)/ZrOx in CO_2 hydrogenation at 400 ^oC.

Supplementary Table 1. Optimization of the Preparation Conditions[a] of $Zr_{0.5}(NiFeCuMnCo)_{0.5}O_x$.

$$ZrCl_4 + NiCl_2 + FeCl_3 + CuCl_2 \bullet 2H_2O + MnCl_2 + CoCl_2 \bullet 6H_2O \xrightarrow[\text{ball-milling,calcination}]{\text{NaCl, NaOH}} Zr_{0.5}(NiFeCuMnCo)_{0.5}O_x$$

Entry	The quality of NaCl (g)	$S_{(BET)}$ (m^2/g)
1	1	65
2	2	68
3	4	84
4	6	83

[a]Reaction Conditions: $ZrCl_4$ (1.165g, 5 eq.), $NiCl_2$ (129 mg, 1 eq.), $FeCl_3$ (162 mg, 1 eq.), $CuCl_2 \bullet 2H_2O$ (170 mg, 1 eq.), $MnCl_2$ (126 mg, 1 eq.), $CoCl_2 \bullet 6H_2O$ (238 mg, 1 eq.), different quality of NaCl, and NaOH (1.24 g, 31 mmol) were mixed by ball milling. Calcination of the as-synthesized powder was performed into a muffle oven in air at 550 ^{o}C for 3 h (2 K min^{-1} to appointed temperature) and then cooled down to room temperature .The powder was washed with deionized water and ethanol, and then put into a vacuum drying oven and dried at 70 ℃ for 12 hours.

Supplementary Table 2. The ICP results of $Zr_{0.5}(NiFeCuMnCo)_{0.5}O_x$.

Element	Wt%	Atomic %
Co	4.672	7.93
Cu	4.922	7.75
Fe	4.394	7.87
Mn	4.234	7.71
Ni	4.091	6.97
Zr	36.27	39.76

Supplementary Table 3. The X values of the as-made materials

Samples	H_2 consumption (mmol g^{-1})	y value	X value
$Zr_{0.5}Co_{0.5}O_{1+y}$	3.17	0.3	1.3
$Zr_{0.5}Ni_{0.5}O_{1+y}$	2.64	0.25	1.25
$Zr_{0.5}Cu_{0.5}O_{1+y}$	2.96	0.29	1.29
$Zr_{0.5}Fe_{0.5}O_{1+y}$	4.31	0.41	1.41
$Zr_{0.5}Mn_{0.5}O_{1.5+y}$	0.39	0.04	1.54
$Zr_{0.5}Mn_{0.25}Cu_{0.25}O_{1.25+y}$	2.66	0.26	1.51

$Zr_{0.5}Co_{0.5}O_{1+y}$, $Zr_{0.5}Cu_{0.5}O_{1+y}$, $Zr_{0.5}Ni_{0.5}O_{1+y}$, $Zr_{0.5}Fe_{0.5}O_{1+y}$: 1 ascribes to unreducible ZrO_2

$Zr_{0.5}Mn_{0.5}O_{1.5+y}$ and $Zr_{0.5}Mn_{0.25}Cu_{0.25}O_{1.25+y}$: 1.5 and 1.25 ascribe to unreducible ZrO_2 and MnO
